# Topic Review: Application of Raman Spectroscopy Characterization in Micro/Nano-Machining

**DOI:** 10.3390/mi9070361

**Published:** 2018-07-21

**Authors:** Zongwei Xu, Zhongdu He, Ying Song, Xiu Fu, Mathias Rommel, Xichun Luo, Alexander Hartmaier, Junjie Zhang, Fengzhou Fang

**Affiliations:** 1 State Key Laboratory of Precision Measuring Technology & Instruments, Centre of MicroNano Manufacturing Technology, Tianjin University, Tianjin 300072, China; hezhongdu@tju.edu.cn (Z.H.); songying@tju.edu.cn (Y.S.); fuxiu@tju.edu.cn (X.F.); fzfang@tju.edu.cn (F.F.); 2 Fraunhofer Institute for Integrated Systems and Device Technology (IISB), Schottkystrasse 10, 91058 Erlangen, Germany; mathias.rommel@iisb.fraunhofer.de; 3 Centre for Precision Manufacturing, Department of Design, Manufacture & Engineering Management, University of Strathclyde, Glasgow G1 1XQ, UK; xichun.luo@strath.ac.uk; 4 Interdisciplinary Centre for Advanced Materials Simulation (ICAMS), Ruhr-University Bochum, Universitätsstraße 150, 44801 Bochum, Germany; alexander.hartmaier@icams.rub.de; 5 Center for Precision Engineering, Harbin Institute of Technology, Harbin 150001, China; junjie.zhang@hit.edu.cn

**Keywords:** Raman spectroscopy, micro/nano-machining, phase transformation, residual stress, TERS

## Abstract

The defects and subsurface damages induced by crystal growth and micro/nano-machining have a significant impact on the functional performance of machined products. Raman spectroscopy is an efficient, powerful, and non-destructive testing method to characterize these defects and subsurface damages. This paper aims to review the fundamentals and applications of Raman spectroscopy on the characterization of defects and subsurface damages in micro/nano-machining. Firstly, the principle and several critical parameters (such as penetration depth, laser spot size, and so on) involved in the Raman characterization are introduced. Then, the mechanism of Raman spectroscopy for detection of defects and subsurface damages is discussed. The Raman spectroscopy characterization of semiconductor materials’ stacking faults, phase transformation, and residual stress in micro/nano-machining is discussed in detail. Identification and characterization of phase transformation and stacking faults for Si and SiC is feasible using the information of new Raman bands. Based on the Raman band position shift and Raman intensity ratio, Raman spectroscopy can be used to quantitatively calculate the residual stress and the thickness of the subsurface damage layer of semiconductor materials. The Tip-Enhanced Raman Spectroscopy (TERS) technique is helpful to dramatically enhance the Raman scattering signal at weak damages and it is considered as a promising research field.

## 1. Introduction

During mico/nano-machining (such as micro/nano cutting, micro/nano milling, micro/nano grinding, etc.) and crystal growth, various defects and damages in crystals occur inevitably, including microstructure change, residual strain, dislocation, phase transformation, and so on [1]. These defects and damages have a significant impact on the mechanical, optical, or electronic performance and sustainability of the machined workpiece [2]. Therefore, they should be reduced and eliminated as much as possible. In fact, if these defects and damages can be accurately characterized by an efficient and powerful method, it will be helpful to understand and deal with them.

Usually, as a typical destructive method, transmission electron microscopy (TEM) is considered as the most direct and effective method to investigate defects and subsurface damages. The subsurface damages such as phase transformation [3], dislocation mobility [4], and even local atom disarrangement [5] can be observed directly by TEM characterization. Other destructive methods include etching [6], cross-sectional observation [7], angle lapping [8], and so on. However, these methods destroy the sample and sometimes the sample preparation is time-consuming, which limits the wide application of these methods. Many non-destructive methods have also been applied to measure and characterize these defects and subsurface damages: the X-ray diffraction technique was adopted by Bismayer et al. [9] to quantitatively determine the residual stress of grinded single crystal silicon. Milita et al. [10] conducted X-ray diffraction to study SiC on insulator structure. The experimental results revealed that quantitative characterization of lattice defects and related stress in a SiC wafer was possible by the X-ray diffraction technique. The photoluminescence technique had been developed and applied to the characterization of a silicon wafer. Buczkowski et al. [11] reported that extended defects (such as dislocations and precipitates) could be imaged by the photoluminescence technique. Nevin et al. [12] pointed out that the photoluminescence technique could be used for detecting and mapping threading dislocations, slip dislocations, and oxide agglomerates. A new laser scattering method was also used to detect subsurface defects. Goto et al. [13,14] measured the depth and dimension of subsurface microdefects in Czochralski-grown silicon wafers and epitaxial silicon wafers using the laser scattering method. They found out that this method could determine the depth of subsurface defects which are less than 5 μm deep, and the boundaries between the epitaxial layers and substrates were detected successfully. However, there are still some problems or shortcomings for the three techniques [15]. (1) For the X-ray diffraction technique, the characterization and identification of phase transformation can not be realized efficiently, and this technology is widely used in powder sample. (2) For the photoluminescence technique, it is mainly applied for the detection of microdefects and dislocations, however, the residual stress detection and phase transformation characterization is hardly feasible. (3) For the laser scattering method, it is a good technique to determine the depth and dimension of defects or damages in the subsurface region, but it can hardly determine the type of defects or damages, which limits its wide application.

As one kind of spectral analysis technique, the Raman effect is based on the inelastic scattering process between the incident photons and the phonons in materials [16]. When the photons of incident light interact with the vibration phonons of the materials, the light is scattered at a frequency and the difference in frequency between the incident and the scattered light provides the information of the lattice vibrations [17]. Therefore, Raman vibration spectra is widely used for providing a structural fingerprint for molecules identification. Besides, the Raman vibration spectra of materials is significantly influenced by microstructural changes, impurities, residual stress, and so on, which leads to changes in the number of scattering molecules, phonon frequency, breakdown of Raman selection rules and other effects [18]. Thus these defects and subsurface damages induced by crystal growth and micro/nano-machining can be characterized using Raman information, namely, band position, band position shift, full width at half maximum (FWHM), and intensity. And best of all, the Raman spectroscopy technique is an efficient, powerful, sample preparation friendly, and non-destructive testing method to characterize these defects and subsurface damages [19].

Given the above information, this review aims to present the principles and applications of the Raman spectroscopy technique in micro/nano-machining. This review is organized as follows. In Section 1, the theory of Raman spectroscopy is introduced, including the principle of Raman spectroscopy, the relationship between incident light wavelength and penetration depth, laser spot size and spectral resolution. In Section 2, typical Raman spectroscopy analysis in the field of micro/nano-machining is introduced, including stacking faults, phase transformation, and residual stress characterization. Finally, the Tip-Enhanced Raman Scattering (TERS) technique is introduced, which shows the significant enhancement for Raman scattering signals and provides a possible method to detect weak damages. 

## 2. Theory of Raman Spectroscopy

In the last decades, Raman spectroscopy technique has been tremendously improved to overcome problems like fluorescence, poor sensitivity, or weak Raman signals. Besides, compared with the conventional dispersive Raman technique, many advanced Raman techniques have been developed to meet the demands of analysis [20]. For example, a Fourier transform (FT) Raman spectrometer using near-infrared (NIR) lasers solves the problem of fluorescence interference [21]. The Surface Enhanced Raman Scattering (SERS) technique produces a large enhancement to the Raman scattering signal [22]. Confocal Raman microscopy can provide three dimensional images of material composition with micrometer resolution and clear image quality [23]. Coherent anti-Stokes Raman scattering (CARS) gives spectral information with excellent sensitivity and low laser power [24]. Resonant Raman Scattering (RRS) allows researchers to explore the material spectra in the range of energies of the photon energy itself (typically 1–3 eV) [25]. In this review, considering the wide applicability, the analysis and discussion about the theory of Raman spectroscopy is restricted to the dispersive Raman technique.

### 2.1. Principle of Raman Spectroscopy

**Figure 1:**
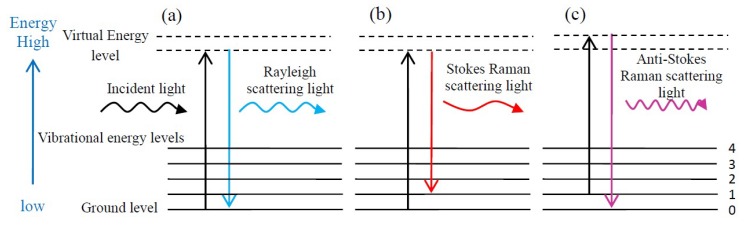
Diagram of the Rayleigh and Raman scattering processes: (**a**) Rayleigh scattering, (**b**) Stokes Raman scattering and (**c**) Anti-Stokes Raman scattering.

The principle of the Raman effect is based on the inelastic light-scattering process between incident light and an irradiated substance [16]. During the light–sample interaction, the incident light interacts with molecules and distorts the electron cloud to form a ‘virtual level’. Since the ‘virtual level’ is not stable, the photons are scattered immediately to another state which is relatively stable [20]. As shown in Figure 1a, when the photons fall back to the initial energy level (in Figure 1a referred to as ground level), there is no energy transfer occurring between the incident light and the scattered light, and there is no change in photon frequency and wavelength. This elastic collision process is known as Rayleigh scattering [20,26]. However, when the photons fall to a new energy level, which is different from the initial energy level, energy transfer happens (i.e., the photon loses or gains some amount of energy) and results in the laser photons energy being shifted down or up, which gives information about the vibrational mode in the system [20,27]. The inelastic scattering of light was predicted theoretically by Smekal [28] in 1923 and firstly observed experimentally by Chandrasekhara Venkata Raman [29] in 1928, which is the reason why the inelastic scattering is called Raman scattering. As shown in Figure 1b,c, Raman scattering can be classified into two types, Stokes Raman scattering and anti-Stokes Raman scattering. In Stokes Raman scattering (Figure 1b), photons are excited from the initial energy level and fall to higher energy level, thus scattered light holds a lower frequency than incident light. In anti-Stokes Raman scattering (Figure 1c), photons are excited from the initial energy level and fall to a lower energy level, thus scattered light holds a higher frequency than incident light. 

The intensity of Raman scattered light is proportional to the number of scattering molecules. The number of molecules in each energy level follows the Boltzmann distribution (Equation (1)) [30].

(1) N1N2=(g1g2)exp[(−ΔE)/KT]

where *N*_1_ and *N*_2_ are the number of molecules in the higher and lower energy level, *g*_1_ and *g*_2_ are the degeneracy of higher and lower energy level, Δ*E* represents the change in energy before and after scattering, *K* is Boltzmann’s constant, and *T* stands for temperature. Thermal equilibrium at room temperature leads to the situation in which the number of molecules in a low energy level vibration is always greater than the number of molecules in a high energy level vibration. The number of molecules in the ground level is the highest, so the Stokes Raman scattering intensity is greater than the anti–Stokes Raman scattering intensity, which is the reason why the Raman scattered light is usually Stokes Raman scattering light (i.e., frequency is lower than the frequency of the incident light).

Raman spectra shows the intensity of scattered light versus wavenumber (the reciprocal of wavelength). The abscissa of Raman spectra (called Raman shift, cm^−1^) is determined by [27],

(2) $$\mathrm{Raman}\;\mathrm{shift}=(\frac{1}{\lambda_{\mathrm{incident}}}-\frac{1}{\lambda_{\mathrm{scattered}}})\times 10^{7}$$

where *λ*_incident_ and *λ*_scattered_ are the wavelengths (nm) of incident light and scattered light, respectively. In particular, the wavenumber is linearly correlated with the energy of incident light and scattered light, which makes the Raman shift of materials usually independent of incident wavelength.

**Figure 2:**
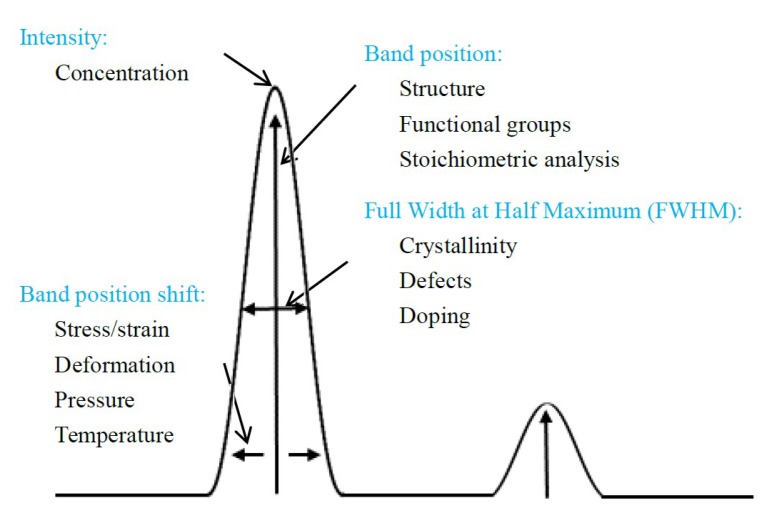
The typical information of Raman spectra and corresponding material information.

During Raman spectroscopy characterization, the Raman spectroscopy instrument collects Raman scattered light from the tested sample and the corresponding Raman spectra carries material information on molecular vibration and crystal structure [31]. Figure 2 shows the typical Raman spectra information and the corresponding material information. Raman spectroscopy has been widely used in various areas such as material science [32,33], chemical industry [34], bio-sensing [35], archeology [36], environment monitoring [37], and so on.

### 2.2. Penetration Depth with Different Laser Wavelengths

Usually, lasers with different wavelengths result in different penetration depths into the sample surface, which allows the information acquisition from different depths by using different laser wavelengths. The shorter laser wavelength gives information closer to the surface. On the other hand, when the selected laser wavelength is too large (or too small), the material information near the surface (or far from the surface) may be concealed (or missed). Thus, choosing the appropriate laser wavelength is very important for obtaining an accurate Raman characterization result, especially for multilayer substrates or surface-sensitive samples, like the ion doping or ion implantation modified semiconductor [38,39].

For Raman spectroscopy, the total scattered light intensity (*I_s_*) integrated from surface to depth (*d*) is expressed as

(3) $$I_{s}=I_{0}D\int_{0}^{d}e^{-2\alpha x}dx=\frac{I_{0}D}{2\alpha }\left(1-e^{-2\alpha d}\right)$$

and the light intensity (*I_d_*) from depth (*d*) to infinity is given by

(4) $$I_{d}=I_{0}D\int_{d}^{\infty }e^{-2\alpha x}dx=\frac{I_{0}D}{2\alpha }e^{-2\alpha d}$$

where *I*_0_, *D*, *α* are the intensity of incident light, the Raman scattering cross section and the photoabsorption coefficient, respectively [40,41]. Assuming that the laser penetration depth *d_p_* is the same as the depth *d*, which meets the relationship of *I_d_*/(*I_s_* + *I_d_*) = 0.1, then the penetration depth *d_p_* can be given by [42,43].

(5) $$d_{p}=\frac{-\ln0.1}{2\alpha }=\frac{2.3}{2\alpha }$$

Given above information, the theoretical penetration depth mainly depends on the absorption coefficient of the material for the different laser wavelengths. For instance, Table 1 summarizes the absorption coefficient and corresponding theoretical penetration depth (according to Equation (5)) of typical semiconductor materials for several commonly used laser wavelengths. Besides, the absorption coefficient and theoretical penetration depth of single crystal SiC and amorphous SiC for different laser wavelengths are listed in Table 2. Based on the calculated laser penetration depth, laser wavelength should be carefully selected during Raman spectroscopy characterization, especially for the micro/nano-scale sensitive surface/subsurface tests. 

### 2.3. Laser Spot Size and Spectral Resolution of Raman Spectroscopy

For a Raman spectroscopy instrument, the laser spot size (spatial resolution) determines the size of the analyzed point, and the results of Raman characterization are unreliable if the laser spot size is larger than the area of interest. Therefore, it is of great importance to determine the appropriate laser spot size before testing. According to the laws of optics, the laser spot size *D* is dependent on the laser wavelength *λ* and the numerical aperture of the microscope objective *NA*. It can be expressed as [46].

(6) D=1.22λNA

where *λ* is the laser wavelength and *NA* is the numerical aperture of the microscope objective. It is clear that a shorter laser wavelength and a larger numerical aperture can provide a smaller laser spot size. 

**Figure 3:**
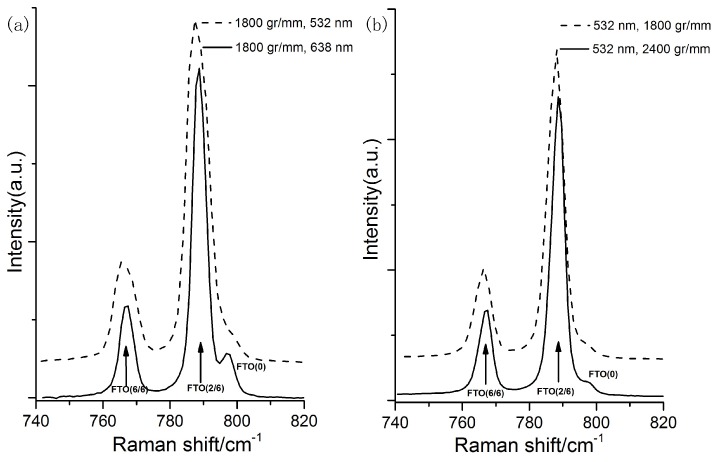
Raman spectra of single crystal 6H-SiC for different spectral resolution induced by: (**a**) different laser wavelengths: 532 nm (1.712 cm^−1^), 638 nm (1.023 cm^−1^); and (**b**) different grating’s groove densities: 1800 gr/mm (1.712 cm^−1^), 2400 gr/mm (1.1378 cm^−1^). (spot size ≈ 1 μm).

Spectral resolution is another significant parameter which determines the accuracy of the obtained Raman spectra. The spectral resolution is dependent on the laser wavelength and the groove density of the spectrometer’s grating. Figure 3a shows Raman spectra (740–820 cm^−1^, using the laser Raman spectroscope XPLORA PLUS from HORIBA Scientific (Minami-ku, Kyoto, Japan) of single crystal 6H-SiC for different spectral resolutions induced by different laser wavelengths (other parameters were strictly controlled and were kept constant). Comparing the spectra for different laser wavelengths, the 638 nm laser wavelength gives a better spectral resolution (1.032 cm^−1^), which makes the details information of the folded transverse optical mode FTO(0) Raman band appear. Likewise, when a grating with a higher groove density is used, the spectral resolution is better and the Raman spectra are finer (as shown in Figure 3b). Both the detailed information of the Raman spectra and the shift of the band position is affected by the spectral resolution. When the spectral resolution is not good enough, the band position shift may not be detected correctly.

## 3. Raman Spectroscopy Characterization in Micro/Nano-Machining

The objective of the present section is to introduce the application of Raman spectroscopy characterization on the defects and subsurface damages induced by micro/nano-machining, which is helpful to characterize the surface integrity. The Raman spectroscopy information is affected by the defects or subsurface damages. Thus, how the defects or subsurface damages affect the Raman spectroscopy information is concerned firstly. Then the applications of Raman Spectroscopy on the stacking faults characterization, phase transformation characterization, and residual stress analysis are introduced. In particular, quantitative calculation of residual stress and the thickness of the amorphous layer are analyzed.

### 3.1. Basis Information

For crystalline materials, Raman scattering can be induced and observed by scattered light which satisfies the following selection rules: (i) energy conservation rule; (ii) wave vector conservation rule, *k*_i_ = *k*_s_ + *q*; and (iii) polarization selection rule, where *k*_i_, *k*_s_, *q* are the wave vectors of incident light, scattered light, and phonon, respectively [47]. Since the wave vector of the phonon contributing to Raman scattering is limited to *q* ≈ 0 when visible laser light is used [48,49], sharp Raman bands are observed in ideal crystals even though there are a number of phonons with different frequencies.

However, this result is not always valid when there are defects or damages in the crystal materials, because defects or damages induce a loss of the periodicity, reduction of symmetry, and disorder of the crystal, which ultimately cause the breakdown of the wave vector selection rule, reduction of phonon lifetime, and reduction of local crystal symmetry [47]. These effects are of great significance for Raman scattering as (1) new Raman bands representing defects can be formed and broadening and asymmetry of the Raman band can also occur due to the breakdown of the wave vector selection rule, (2) broadening of the Raman band will be induced by the reduction of phonon lifetime, (3) the reduction of the local crystal symmetry changes the polarization selection rule and allows the observation of the Raman band at a Raman forbidden geometry [47].

The strain/stress and Raman shift are closely related. As mentioned before, the Raman effect is produced when photon energy is increased or decreased owing to photons scattering from molecules or crystal lattices and it reflects the lattice vibration energy of the material in terms of Raman shift. When the material is strained, the crystal structure (or energy level) of the material is altered, thus the Raman shift will be changed [50].

Besides, the defects inside the material also have an impact on the intensity of Raman bands. It is well known that Raman scattering is induced by the inelastic collision between the incident photons and the phonons in the material. The intensity of Raman scattered light is proportional to the number of scattering molecules irradiated by the incident light and inversely proportional to the absorption coefficient of the material. With an increase in defects or damage concentration inside crystalline material, the number of scattering defects (or damages) and the absorption coefficient of the material increase. The former leads to intensity increasing of the Raman bands caused by defects [47,51], while the latter induces a reduction of the crystalline phase Raman band [51,52].

### 3.2. Stacking Faults Characterization

**Figure 4:**
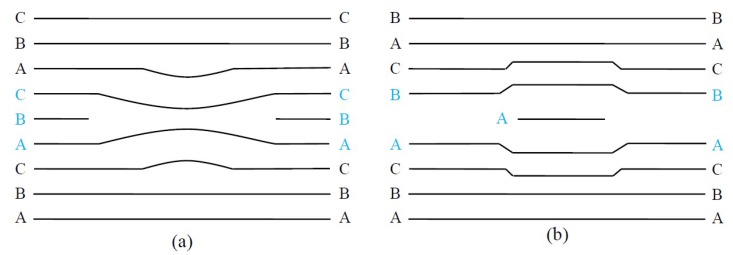
Stacking faults in semiconductors: (**a**) intrinsic stacking fault; and (**b**) extrinsic stacking fault.

Stacking faults are one of the typical planar defects, in which the stacking periodicity of the atom planes is interrupted. As shown in Figure 4, when a part of an atom plane is missing, this is called the intrinsic stacking fault, and the extrinsic stacking fault means an extra atom plane being present [53]. Since there are a large number of polytypes with different stacking sequences of Si-C double atomic planes [54] and the difference in the formation energies of individual polytypes is small [55], stacking faults are easy to generate and they are typical defects in SiC crystals. It has been proved that stacking faults have a great influence on the electrical properties of SiC [56,57] and high-density stacking faults can enhance the radiation resistance of SiC by more than an order of magnitude [58]. So it is of great importance to accurately characterize stacking faults in SiC.

It is well known that all atoms in solids hold in their equilibrium position by the force which holds the crystal together. When the atoms are displaced from their equilibrium positions, they are affected by restoring forces, and vibrate at characteristic frequencies. These vibrational frequencies are determined by the phonon modes of the crystal. For any crystal, the number of phonon modes is 3N, where N is the number of atoms per unit cell. Out of these 3N modes, three are acoustic (A) modes and 3N-3 are optical (O) modes. Also, they are usually classified into transverse (T) mode (the atomic displacement is perpendicular to the direction of wave travel) and longitudinal (L) mode (the atomic displacement is parallel to the direction of wave travel). For example, transverse optical (TO) modes are referred to as TO modes, and longitudinal optical (LO) modes are referred to as LO modes. However, not all of the modes are active in Raman scattering [59]. These modes are Raman active or ‘allowed’ in the Raman scattering only when the vibrations are associated with the change of polarizability. Otherwise, the modes are not allowed, they are ‘forbidden’ in the Raman scattering [60].

**Figure 5:**
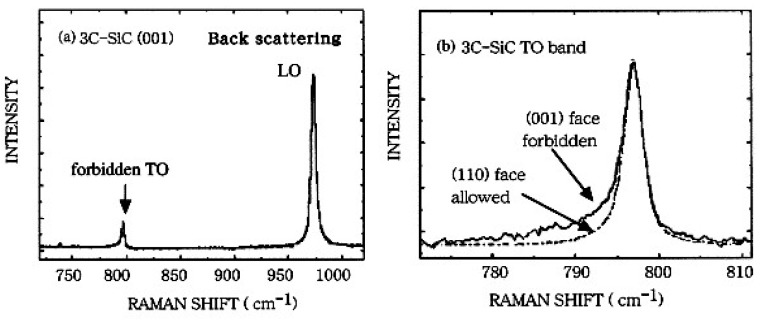
(**a**) Raman spectra of a 3C-SiC at the back scattering geometry using (001) face, (**b**) Comparison of the TO band, the dashed line and solid line are spectra measured using the (110) face and (001) face, respectively. Reproduced with permission from Elsevier [61]. (spot size ≈ 1.32 μm, 588 nm laser wavelength).

Figure 5 shows the Raman spectra of 3C-SiC layer, which was grown on Si (001) and contained stacking faults, dislocations, and antiphase boundaries at around the interface of SiC and Si. Using the (001) face, the TO and LO modes are Raman forbidden and active, respectively, while the TO and LO bands are Raman active and forbidden using the (110) surface [61]. Figure 5a shows that the intensity of the TO band was very weak at forbidden geometry. As shown in Figure 5b, the shape of the forbidden band at the high frequency side was almost the same as that of the allowed band, which indicated that the asymmetric TO band resulted from the breakdown of the wave vector and polarization selection rules induced by the stacking faults. Besides, broadening of the forbidden TO band at the high frequency side was found, which revealed that the defects and stacking faults shorten the phonon lifetime [61].

For *n*H-SiC and 3*n*R-SiC polytypes materials, the folded modes observed in the Raman spectra correspond to phonons with a wave vector of *q* = 2π*m*/(*nc*) in the basic Brillouin zone of the 3C-polytype in the [111] direction, where *n* and m are integers (2*m* ≤ *n*) and c is the unit-cell length of the 3C-polytype along the [111] direction [49]. The folded modes for *n*H-SiC and 3*n*R-SiC polytypes includes folded transverse optical mode (FTO(*x*)), folded transverse acoustic mode (FTA(*x*)), folded longitudinal optical mode (FLO(*x*)), and folded longitudinal acoustic mode (FAO(*x*)), where the numbers *x* in parentheses represent reduced wave vector and *x* = 2*m*/*n* [62,63].

**Figure 6:**
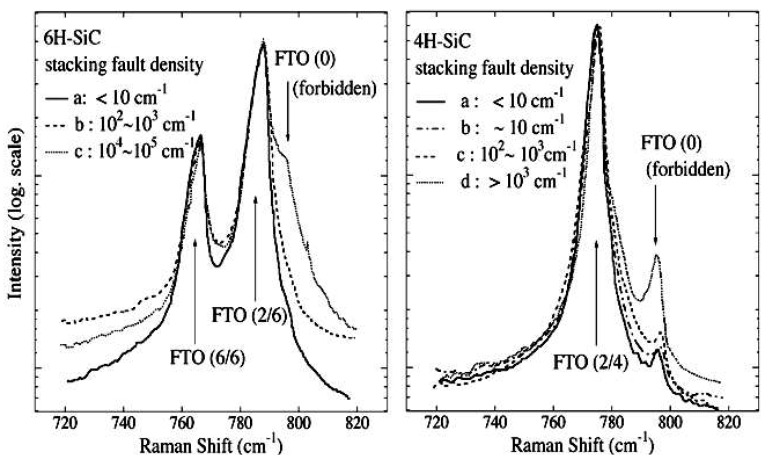
Raman spectra of 6H-SiC (0001) crystals and 4H-SiC (0001) crystals with different stacking faults densities. Reproduced with permission from Springer Nature [47]. The samples with different densities of stacking faults were cut from 4H- and 6H-SiC ingots and the stacking faults densities were evaluated by etch-pit-density measurements. (spot size ≈ 1.32 μm, 588 nm laser wavelength).

Figure 6 shows Raman spectra of 6H-SiC and 4H-SiC crystals with different stacking fault densities [47]. A new Raman band at 796 cm^−1^ (FTO(0)) with high densities of stacking faults in both 4H-SiC and 6H-SiC appeared in the spectra. The intensity of the FTO(0) band increased with the stacking fault density, while the FTO(2/6) and FTO(6/6) bands in 6H-SiC hardly changed, which was the same as for the 4H-SiC sample (no remarkable change was observed for FTO(2/4)). For perfect 6H-SiC (and 4H-SiC) crystals, the Raman bands of FTO(2/6) and FTO(6/6) (and FTO(2/4)) are Raman active, while the FTO(0) band is Raman inactive in the back scattering configuration using the (0001) face [61,64]. The stacking faults in the SiC crystals induced the breakdown of the wave vector selection rule [65,66], so the Raman forbidden band of FTO(0) was produced and the growth of this Raman band could be explained based on the bond Raman polarizability model [47,64]. Given the above information, the FTO(0) mode was activated by stacking faults and this mode could be used as a monitor of stacking faults density. On the contary, the FTO(2/6) and FTO(6/6) (and FTO(2/4)) bands were almost independent of the stacking faults density.

### 3.3. Phase Transformation Characterization

Phase transformation during or after micro/nano-machining has been widely reported, including micro/nano-cutting [67], micro/nano-indentation [4], micro/nano-scratching [5], Focused Ion Beam (FIB) machining [68] and so on. Phase transformation characterization and identification is of great significance for understanding material removal mechanism.

**Figure 7:**
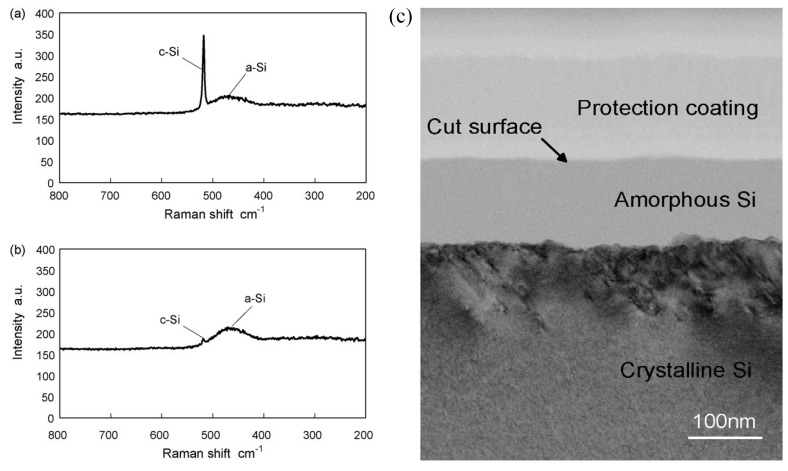
Raman spectra of surface regions machined under different depths of cut: (**a**) 5 nm and (**b**) 120 nm (**c**) transmission electron microscopy (TEM) micrographs at depths of cut of 120 nm. Reproduced with permission from Elsevier [52]. (laser spot size was 1 μm, the laser wavelength was 532 nm).

**Figure 8:**
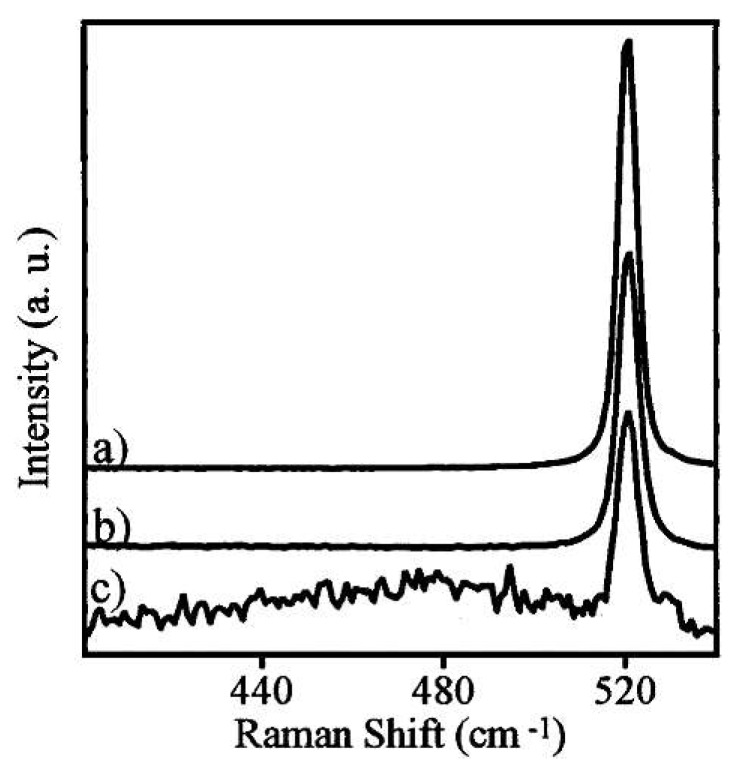
Raman spectra of the silicon: (**a**) before machining; (**b**) after machining in brittle mode; and (**c**) after machining in ductile mode. Reproduced with permission from Springer Nature [71]. (performed with the 488 nm exciting light, the spot size was sufficiently small compared with the dimensions of the machined surface).

For crystalline Si (c-Si), the first-order Raman spectra displays a sharp peak at the Raman shift of 520 cm^−1^ with a natural full width at half maximum (FWHM) of ≈3.5 cm^−1^ at room temperature, which is induced by triple degenerate optical phonons [69]. For amorphous silicon (a-Si), an optical band peak at 470 cm^−1^ is displayed in the first order Raman spectra [69,70]. At maximum disorder (i.e., a-Si), owing to the breakdown of the wave vector selection rule induced by the loss in long range order, all phonons are therefore optically allowed and the Raman spectra resembles the phonon density of states with a new broad Raman band at 470 cm^−1^ [69]. Figure 7 shows typical Raman spectra of the crystalline peak and amorphous peak of silicon at 520 cm^−1^ and 470 cm^−1^, respectively [52]. The Raman spectra of c-Si and a-Si can be used as the reference when studying the Raman spectra from silicon with subsurface damages. Pizani et al. [71] used Raman spectroscopy to measure the sample surface machined by single point diamond turning of silicon. As shown in Figure 8, compared with the non-machined surface, a new Raman broad band at about 470 cm^−1^ was detected in ductile machining (Figure 8c), which was attributed to the optical band of a-Si resulting from a thin amorphous layer at the sample surface. Besides, the lack of any feature at about 470 cm^−1^ in the brittle machining area indicated the absence of an amorphous phase (Figure 8b).

**Figure 9:**
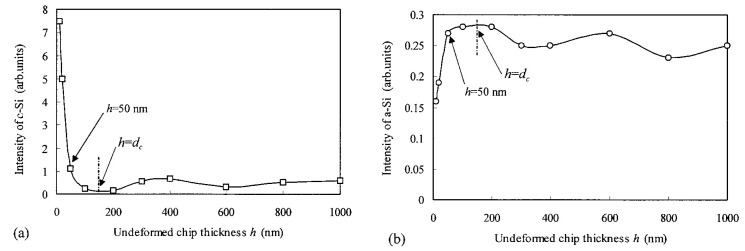
Raman intensities of (**a**) crystalline phase (c-Si) and (**b**) amorphous phase (a-Si) with respect to undeformed chip thickness during single point diamond machined Si substrates. Reproduced with permission from AIP Publishing [51]. (laser spot size: 1 μm, the laser wavelength: 514.5 nm).

In addition, the intensity information of Raman scattering is also useful to analyze the phase transformation. Yan [51] used laser Micro-Raman spectroscopy to examine the machined surface by single point diamond turning of Si with different underformed chip thicknesses. Figure 9 shows the intensities of the crystalline and amorphous phases Raman peak with respect to the underformed chip thickness. The results revealed that the minimum intensity of the crystalline phase and the maximum intensity of the amorphous phase were both obtained near the ductile–brittle transition boundary (*d*_c_ ≈ 150 nm) and the thickness of the amorphous damage layer (*d*_a_) increased with the underformed chip thickness during ductile removal.

**Figure 10:**
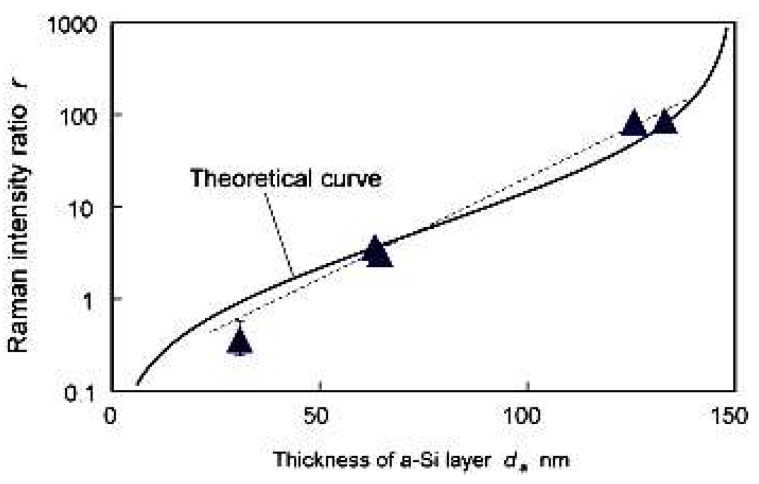
Theoretical relationship and experimental results of Raman intensity ratio and amorphous layer thickness induced by micro/nano-machining. Reproduced with permission from Elsevier [52].

Further, an analytical prediction model was proposed by Yan et al. [52] to quantitatively calculate the thickness of the subsurface amorphous layer induced by micro-nano machining using Raman spectroscopy. They proposed a new parameter named Raman intensity ratio *r* based on Raman spectra. The relationship between the thickness of the amorphous layer *d_a_* and the Raman intensity ratio *r* was

(7) $$d_{a}=33.3\times \ln\{\frac{8.84+15r}{8.84+0.167r}\}$$

where *d_a_* was the thickness of the amorphous phase, *r* was the Raman intensity ratio, $r=\frac{I_{a}}{I_{c}}$, *I_a_* was the total Raman intensity of the amorphous silicon, and *I*_c_ was the total Raman intensity of the crystalline silicon. Figure 10 shows the theoretical curve in comparison with experimental results, which revealed the possibility of quantitatively calculating the thickness of subsurface damages layer by Raman spectroscopy information.

**Figure 11:**
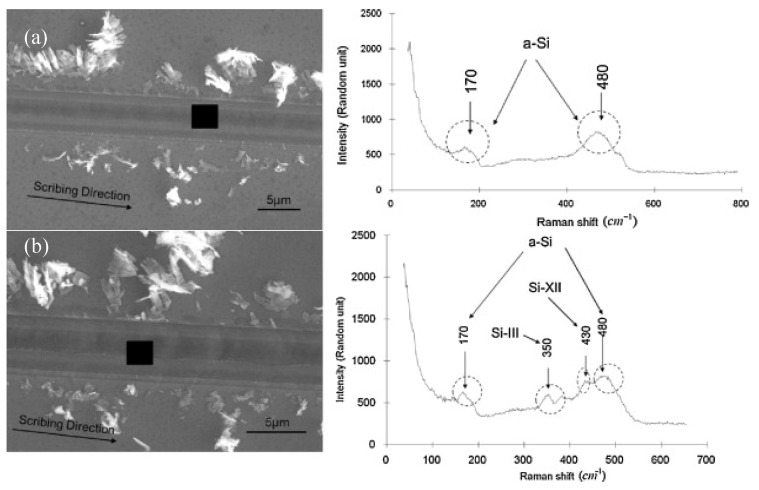
Raman spectra of c-Si samples which were diamond-scribed along (110) [001] under different scribing speeds: (**a**) 5 mm/min, (**b**) 1 mm/min. Reproduced with permission from Elsevier [74]. (the black square in the SEM image indicates the analyzed points).

**Figure 12:**
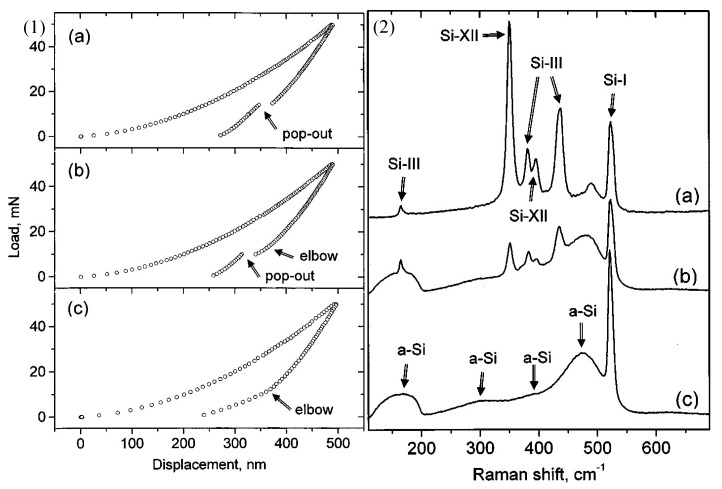
(1) Three typical load–displacement curves in the nano-indentation of Si (loading rate of 3 mN/s and maximum load of 50 mN), (2) Corresponding Raman spectra of the nano-indentations: (**a**) metastable phases, (**b**) a mixture of a-Si and metastable phases, (**c**) amorphous Si. Reproduced with permission from AIP Publishing [76]. (spot size ≈ 1 μm, 514.5 nm Ar^+^ laser).

High pressure phase transformation (HPPT) is of great significance to the ductile removal mechanism of hard and brittle materials [72], such as Si and SiC. Since different new phases (crystal structure) may be generated due to HPPT during micro/nano-machining [73], identifying these new phases (crystal structure) is significant for understanding the mechanism of machining. Raman spectroscopy can not only characterize the amorphous phase (as above), but also identify various phase species (crystal structure). Raman spectroscopy characterization had been performed by Wu et al. [74] to study the ductile response of c-Si after scribing with different scribing speeds. Figure 11 shows the analyzed area (the black square) and corresponding Raman spectra obtained from the ductile portion of the track after diamond scribing in (110) [001] c-Si under different scribing speeds. The 480 cm^−^^1^ and 170 cm^−^^1^ broad Raman bands corresponded to a-Si, and the 430 cm^−1^ and 350 cm^−^^1^ Raman bands represent the Si-XII and Si-III phases, respectively [75]. As shown in Figure 11, the metastable phases Si-XII and Si-III were observed for a scribing speed of 1 mm/min, while there were only a-Si bands visible for 5 mm/min, which indicated that a low scribing speed more likely led to the formation of crystalline phases (the low scribing speed corresponds to the low unloading rate, which more likely leads to the formation of crystalline phases). Domnich et al. [76] carried out nanoindentation tests in Si (111) and Si (100) using a Berkovich pyramid. Peaking loading was 30–50 mN and the loading rates ranged from 1 to 3 mN/s. Raman spectra were acquired in the backscattering geometry. Figure 12 shows three typical load–displacement curves and corresponding Raman spectra. A strong correlation between the shape of the unloading curve and phase transformations occurrences was revealed. The pop-out effect showed an abrupt phase transformation from metallic Si-II to either Si-III or Si-XII, and it was accompanied by a sudden increase in volume. The elbow corresponded to the formation of an amorphous phase of silicon [76]. For single crystal Si, the crystalline structure (Si-I) contains four nearest neighbours at an equal distance of 2.35 Å at ambient pressure [72]. During micro/nano-machining, the cutting edge radius or negative effective rake angle result in large hydrostatic pressure in the cutting zone [77,78]. Under hydrostatic pressure of 10–12 GPa, Si-I transforms to the Si-II phase, which contains four nearest neighbours at a distance of 2.42 Å and two other neighbours at 2.585 Å [79]. The reverse transformation from the Si-II phase might occur during releasing the pressure (unloading). Under quick unloading, the Si-II phase directly transforms into the amorphous phase. However, the crystalline phases of Si-XII (four nearest neighbours at a distance of 2.39 Å and another at a distance of 3.23 Å) and Si-III (four nearest neighbours at a distance of 2.37 Å and another atom at a distance of 3.41 Å) may be generated under slow unloading [72]. These new crystal structures (i.e., Si-II, Si-III, and Si-XII phase) generated during the micro/nano-machining have a new lattice vibrational frequency, so the corresponding sharp Raman bands can be observed during Raman characterization.

**Figure 13:**
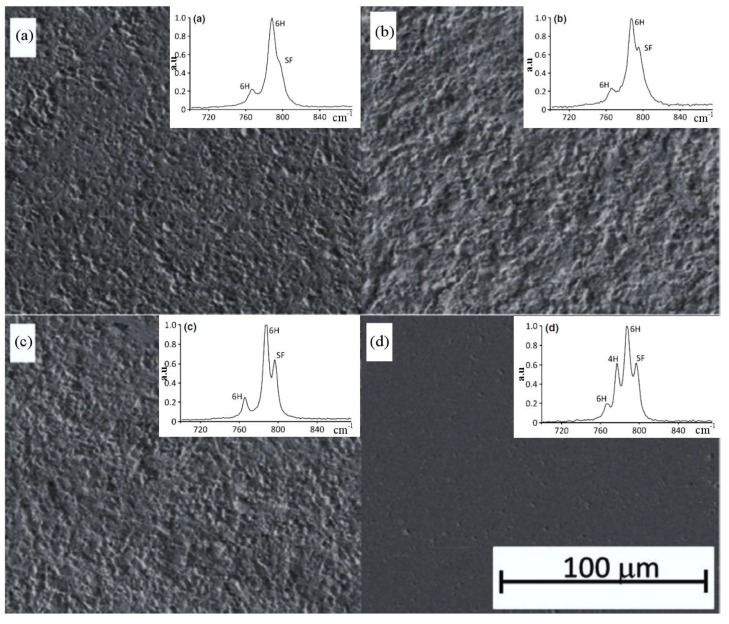
The machined surface and corresponding typical Raman spectra of single crystal 6H-SiC under different machined surface finishes: (**a**) standard finish, (**b**) grit blast, (**c**) rotary ground, and (**d**) mirror finish. Reproduced with permission from John Wiley and Sons [80]. (40 μm × 40 μm areas of the machined surfaces were scanned point by point with the individual points spaced 2 μm apart, the spot size was smaller than 1 μm).

Raman spectroscopy has also been widely used in the identification of phase transformation for silicon carbide. Single crystal 6H-SiC under different mechanical machining methods was analyzed using Raman spectroscopy, as shown in Figure 13 [80]. The standard finish was similar to the non-machined surface. As shown in Figure 13, the different Raman bands of the different crystal structures (i.e., 6H-SiC, 4H-SiC and stacking faults) were characterized successfully. The scratching of 4H-SiC was also analyzed using Raman spectroscopy by Nakashima et al. [81]. After scratching, the broadening and/or asymmetry of the FTO (1/2) (776 cm^−1^) and LO (964 cm^−1^) modes and the downshift of the FTO (1/2) mode were observed. They claimed that the asymmetry downshift of the Raman band might be originated from stacking faults and/or residual strain, and the broadening indicated the generation of defects (including dislocations) during scratching. Not only the above machining methods, but also lapping [17], ion implantation [82,83], and others had also adopted Raman spectroscopy to identify phase transformation successfully. 

### 3.4. Residual Stress Analysis

Residual stress within the subsurface of a mechanically machined workpiece is widely reported and the residual stress can profoundly influence the mechanical properties of the workpiece, such as strength and toughness [84]. Determination of the residual stress can provide very important information to improve the mechanical properties of the workpiece. Fortunately, Raman spectroscopy is very useful for detecting residual stress in single crystal and polycrystalline materials [50].

It was as early as the 1970s that the sensitivity of the Raman shift to mechanical stress was first reported by Anastassakis et al. [85]. Further, Ganesan et al. [86] showed that the band position shift was sensitive to the strain and it could be expressed in terms of material constants. Subsequently, according to experimental results, Weinstein and Piermarini [87] proposed an equation to relate the band position of the first-order Raman band of Si with stress:

(8) ω(cm−1)=519.5±0.8 cm−1+(0.52±0.03 cm−1)σ+(−0.0007±0.0002 cm−1)σ2

where *ω* was the measured position of the characteristic crystalline band of Si, *σ* was the stress within the machined surface, and its unit was kbar (1 kbar = 0.1 GPa).

In 1996, based on the theoretical derivation, Wolf [43] revealed that for back scattering from the Si (001) surface, the relation between the band position shift of a crystalline Si Raman band and residual stress could be determined by the following equation: 

For uniaxial stress,

(9) $$\Delta \omega ({\mathrm{cm}}^{-1})=-2\times 10^{-9}\sigma (\mathrm{Pa})$$

For biaxial stress in the *x*–*y* plane, with stress components *σ_xx_* and *σ_yy_*,

(10) $$\Delta \omega ({\mathrm{cm}}^{-1})=-4\times 10^{-9}\left(\frac{\sigma_{xx}+\sigma_{yy}}{2}\right)(\mathrm{Pa})$$

where $\Delta \omega =\omega_{ref}-\omega_{sample}$ was the band position shift between the measured Si Raman band and the band position of a non-stressed sample at 520 cm^−1^. In addition, they pointed out that a compressive uniaxial or biaxial stress would result in an increase of the Raman shift, while tensile stress would cause a decrease. 

Further development regarding the quantitative calculation of Si Raman peaks with equal biaxial stress revealed that the band position shift with respect to the Si crystalline band follows [50]:

(11) $$\Delta \omega \left({\mathrm{cm}}^{-1}\right)=-4.00\times 10^{-10}\sigma$$

where *σ* was in dyne/cm^2^ (1 dyne/cm^2^ = 0.1 Pa) and the Raman shift for unstressed single crystal Si was 520.28 cm^−1^ with an uncertainty of 0.06 cm^−1^.

**Figure 14:**
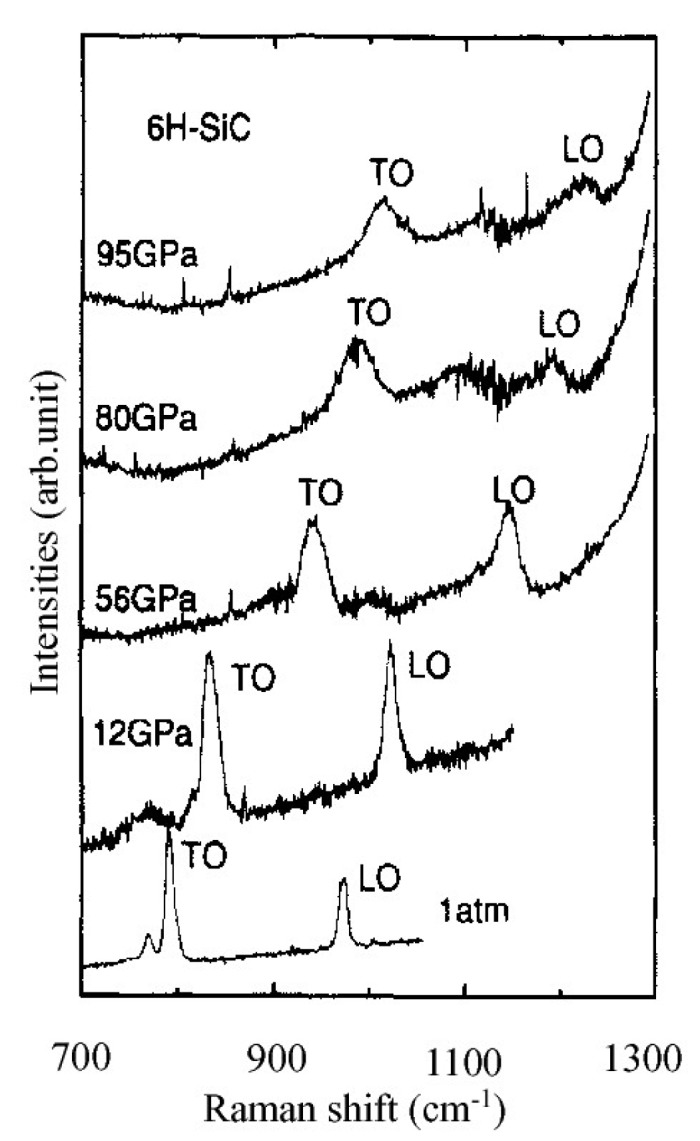
Raman spectra of 6H-SiC samples at different pressures. Reproduced with permission from American Physical Society [88]. The pressure was applied and measured by a diamond anvil cell device and ruby fluorescence, respectively. (spot size ≈ 5 μm, 514.5 nm Ar^+^ laser).

Silicon carbide materials had also been widely studied for quantitative calculation of residual stress using Raman spectroscopy [88,89,90,91,92]. Figure 14 shows typical Raman spectra of the LO and TO modes at different pressures obtained from a single crystal 6H-silicon carbide [88]. It was obvious that the Raman shift of the LO and TO modes was related with pressure and increased with increasing pressure. Besides, DiGregorio et al. [90] suggested that the Raman shift of the TO mode and LO mode of SiC materials and the residual stress met the following equation:

(12) $$\begin{array}{l} \omega^{\mathrm{TO}}-\omega_{0}^{\mathrm{TO}}\left({\mathrm{cm}}^{-1}\right)=-\left(3.53\pm 0.21\right)\sigma \\ \omega^{\mathrm{LO}}-\omega_{0}^{\mathrm{LO}}\left({\mathrm{cm}}^{-1}\right)=-\left(4.28\pm 0.22\right)\sigma \end{array}$$

where $\omega^{\mathrm{TO}}$ and $\omega_{0}^{\mathrm{TO}}$ were the Raman shift of the TO mode with and without residual stress, respectively, $\omega^{\mathrm{LO}}$ and $\omega_{0}^{\mathrm{LO}}$ were the Raman shift of the LO mode with and without residual stress, respectively, *σ* was the measured residual tensile stress in GPa. This had been proven to be consistent for *α* and *β*-SiC [93,94,95]. For 3C-SiC (*β*-SiC), the TO mode and LO mode for the unstressed state are 796 cm^−1^ and 973 cm^−1^, respectively [96]. For single crystal 6H-SiC, the residual stress measurement was realized using Raman spectroscopy by Zingarelli et al. [89] and they concluded that the Raman shift *ω* of TO1 (766 cm^−1^) and TO2 (788 cm^−1^) mode could be determined by [89]:

(13) $$\begin{array}{l} \omega_{\mathrm{TO}1}\left({\mathrm{cm}}^{-1}\right)=-1.62\sigma +766.6\\ \omega_{\mathrm{TO}2}\left({\mathrm{cm}}^{-1}\right)=-3.02\sigma +788.4 \end{array}$$

where *σ* was a positive tensile pressure measured in GPa.

With the development of the Raman spectroscopy, in particular, since the spatial resolution of Raman spectroscopy is less than 1 μm (micro-Raman spectroscopy, μ-Raman), Raman spectroscopy has become an important tool for studying local stress and it has been applied more and more in detecting and calculating quantitatively residual stress on the basis of the band position shift of Raman spectra [97,98,99]. For example, in order to compare the residual stress induced by micro-machining of single crystal Si using femtosecond and nanosecond lasers, micro-Raman spectroscopy detection was performed by Amer et al. [98]. The residual stress was calculated using the information of band position shift. The results revealed that the residual stress depended on the laser fluence and reached a maximum at around 50 J·cm^−2^ (2.0 GPa) and 25 J·cm^−2^ (1.5 GPa) for nanosecond and femtosecond lasers, respectively. Besides, the residual stress caused by diamond turned single crystal Si was also calculated using Raman shift by Jasinevicius et al. [97]. They concluded that the residual stress decreased with the feed rate up to 2.0 μm/rev and then tended to be constant with further increase of feed rate. Also, the residual stress decreased with the increase in the cutting depth. The residual stress induced by nanosecond laser drilling of Si was calculated using Equation (9), and the unstressed position of Si was 520 cm^−1^ [99]. The results revealed that residual stress depended on the diameter and polarization of the laser beam.

**Figure 15:**
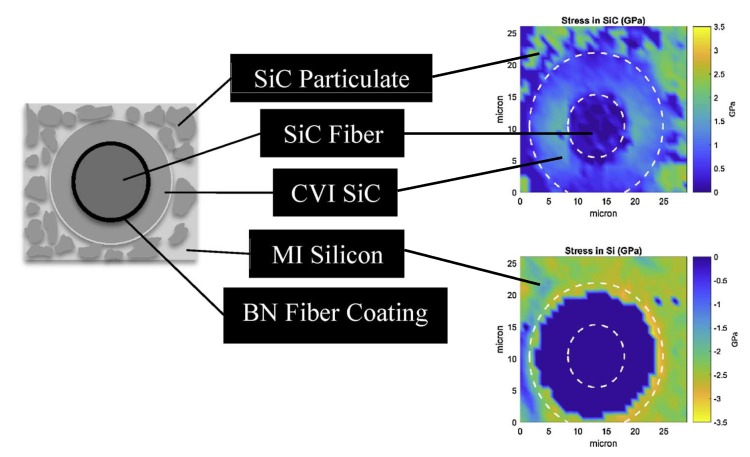
Residual stresses of silicon carbide and unreacted silicon around the Sylramic fibers measured by micro-Raman spectroscopy in a 30 μm × 30 μm map. Reproduced with permission from Elsevier [101]. (30 μm × 30 μm area of the machined surfaces was scanned point by point with the individual points spaced 1 μm apart, the spot size was smaller than 1 μm, 514.5 nm Ar^+^ laser).

The development of new generation Raman microspectrometers (efficiency increased dramatically) makes Raman mapping available as a new technique [100]. The detected area is scanned point by point with the laser and the Raman spectra in each point are collected. Figure 15 shows Raman mapping results of residual stress formed by the melt infiltrated process around Sylramic fiber for both SiC and Si (CVI: chemical vapor infiltrated, MI: melt-infiltrated, BN: boron nitride) [101]. Based on the band position shift, the collected Raman data was imported into a MATLAB program to calculate the residual stress maps of the detective area. The calculation process was applied using Equations (9) and (11) for Si and SiC, respectively, and the corresponding reference Raman shifts (without residual stress) were 515.3 and 796 cm^−1^ (TO mode). It was noted that due to the presence of boron doping in the silicon, the reference Raman shift (without residual stress) of silicon (≈520 cm^−1^) could not be used and the reference Raman shift (515.3 cm^−1^) was determined experimentally.

## 4. Future Prospects

In the previous sections, this review paper provides the information about the application of Raman spectroscopy in micro/nano-machining, which is helpful to ensure the surface integrity. However, Raman scattering is so weak that only one in 10^6^−10^8^ scattered photons carries the Raman signal, which makes it more difficult to detect the damages or defects signals when the damages or defects are too weak, especially for the damages or defects induced by nanometric machining. Therefore, during characterizing the micro/nano-machined workpiece, the approaches on how to enhance the Raman signals for the weak damages or defects are an important research field.

**Figure 16:**
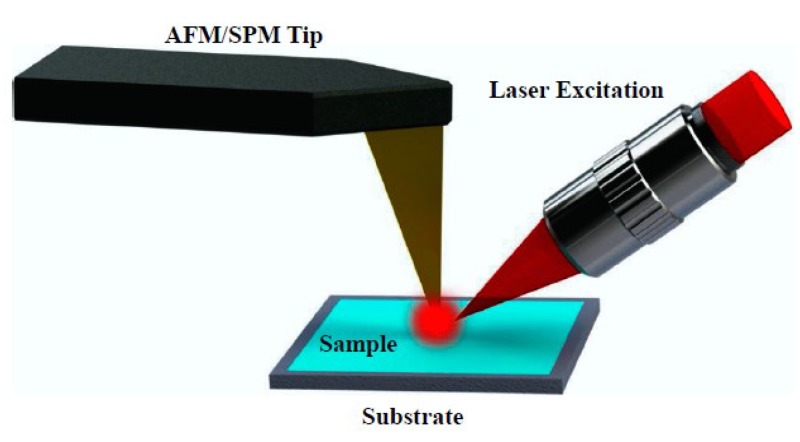
Schematic of a Tip-Enhanced Raman Spectroscopy device which combines an AFM/SPM Tip and a Laser Excitation. Reproduced with permission from Springer Nature [103].

By a combination of the Atomic Force Microscopy (AFM)/Scanning Probe Microscopy (SPM) technique and Raman spectroscopy technique, the TERS is a rapidly developing method in recent years, which provides a higher resolution and enhanced Raman signal [102]. As shown in Figure 16, TERS measurement requires a modified AFM or SPM tip and a Raman laser excitation [103]. When the tip is modified with plasmonic material such as Ag or Au by electrochemical etching or physical metal deposition, an enhanced and localized electromagnetic field will be generated in a few nanometers area at the tip-surface junction [104]. Similar to the SERS technique, both electromagnetic effect and chemical effect contribute to the TERS enhancement while electromagnetic effect is the dominant contributor [104]. Further, the incident light excites the coupling of sample surface plasmon and nanoscale sharp tip plasmon, thus the enhanced Raman signal and optimized spatial resolution are generated [104].

**Figure 17:**
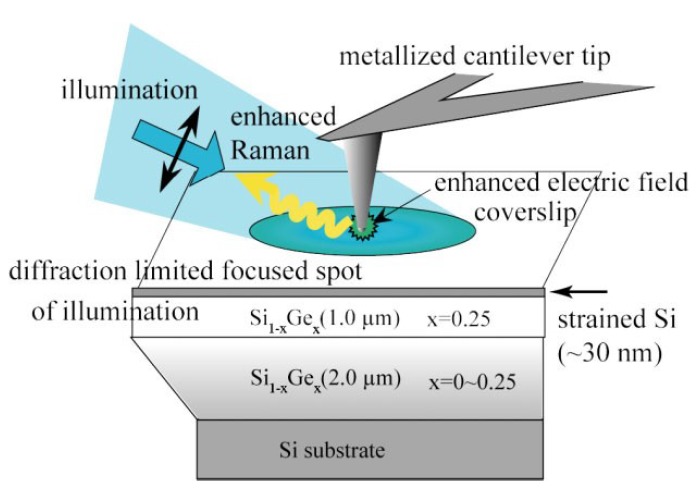
Schematic of TERS microscopy for thin strained Si layer. Reproduced with permission from John Wiley and Sons [105].

**Figure 18:**
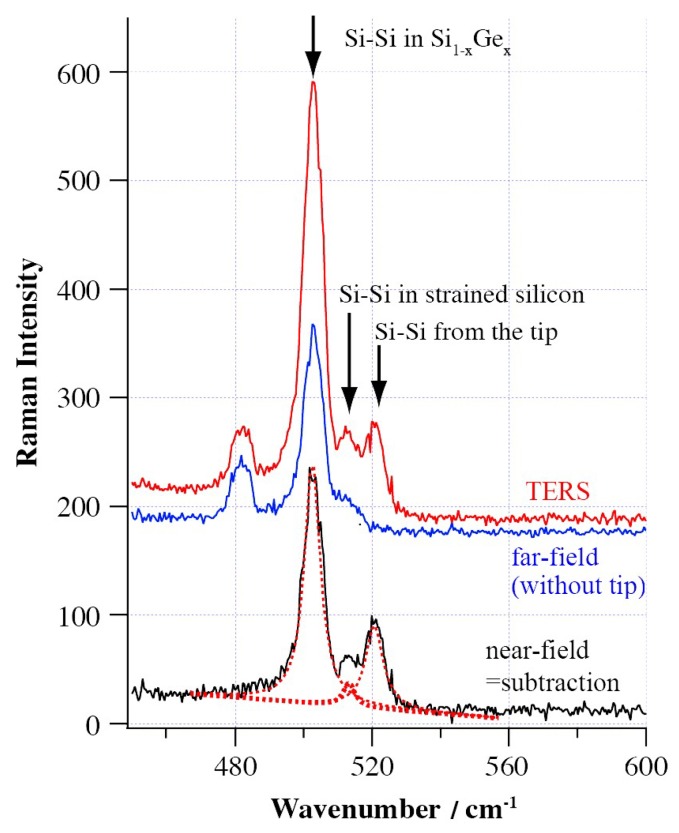
TERS spectra (=with tip, red solid), far-field Raman spectra (=without tip, blue solid) and the near-field spectra (=subtracted, black solid) of 30nm strained Si layer. Reproduced with permission from John Wiley and Sons [105].

For micro/nano-machining (particularly for nanometric machining), sometimes the thickness of the damages layer is very small (nanoscale) and the conventional Raman damages signals can not be collected efficiently. This problem can be solved by utilizing the TERS technique, which can efficiently obtain the near-field signal from the small volume around the tip apex when the tip is close enough to the surface of thin damaged layer. Hayazawa et al. [105] detected the weak strain Raman signals orginating from a nanometric scale thin layer (≈30 nm) utilizing the TERS technique. As shown in Figure 17, the strained layer was fabricated by Ge doping in Si substrate (≈30 nm). The 2 μm-thick bottom layer had an increased concentration of Ge up to 25% and the 1 μm-thick medium layer had a constant Ge concentration of 25%. Figure 18 shows the TERS spectra (with tip), far-field Raman spectra (without tip), and the near-field spectra (subtracted). The far-field signal was obtained without tip and it had a significant band of Si-Si in Si_1−*x*_Ge_*x*_, but no obvious Si-Si band from strained Si layer. The TERS signal could clearly distinguish the Si-Si band (520 cm^−1^) from tip, strained Si layer (514 cm^−1^), and Si_1−*x*_Ge*_x_* buffer substrate (503 cm^−1^). After subtracting the far-field background signal, a background-corrected spectrum was obtained which contains the detailed localized information of the strained layer from the region around the tip apex [105]. Besides, Zhu et al. [106] also adopted the TERS technique to realize the calculation of residual stress, which was attributed to the strained silicon film of 70 nm thickness.

Overall, with the help of the TERS technique, it is possible to enhance the Raman scattering signal. Also, considering the high performance of TERS in the characterization of a weakly damaged layer, the TERS technique is thus execpted to make a great contribution to the field of micro/nano-machining (i.e., defects and subsurface damages characterization) in the future.

## 5. Conclusions

In this paper, we reviewed the fundamentals and applications of Raman spectroscopy for the characterization of defects and subsurface damages in micro/nano-machining. The principle of Raman spectroscopy was introduced and it was shown that Raman spectra information could effectively reflect crystal structure information of materials. Three critical parameters (i.e., penetration depth, laser spot size, and spectral resolution) of Raman spectroscopy were discussed. Raman spectroscopy characterization of stacking faults, phase transformation, and residual stress in micro/nano-machining was reviewed in detail.

Defects and subsurface damages in crystalline materials induce a breakdown of the wave vector selection rule, a reduction of phonon lifetime, and a reduction of local crystal symmetry, which makes the Raman spectra information different from that obtained for perfect crystal. For single crystal 4H-SiC and 6H-SiC materials, the FTO(0) Raman band is a monitor of stacking faults, that is, the higher the stacking faults density is, the larger the intensity of the FTO(0) Raman band will be. The feasibility of the Raman spectroscopy technique for characterizing and identifying phase transformation induced by micro/nano-machining has been demonstrated on semiconductor materials and the presence of new phases on the machined surfaces is clearly shown. Besides, for single crystal Si, it is possible to calculate the thickness of the subsurface damage layer by Raman spectroscopy. Also, the relationship between residual stress and the band position shift is revealed for Si and SiC, which makes it feasible to calculate quantitatively the residual stress using Raman spectroscopy. 

Measuring the weak Raman scattering signal at low damages remains a theoretical and technical challenge. With the help of the TERS technique, it is possible to dramatically enhance the Raman scattering signal and it is considered as an alternative method to solve the problem.

## Figures and Tables

**Table 1:** The absorption coefficient (10^−3^α, cm^−1^) [44] and penetration depth (*d_p_*, nm) (calculated using Equation (5)) of the typical semiconductor materials under different laser wavelengths (nm) and corresponding photon energy (hυ (eV), where h is Planck’s constant).

**Category**	457.9 nm		488.0 nm		514.5 nm		532.0 nm		638.0 nm	
	2.710 eV		2.542 eV		2.410 eV		2.332 eV		1.945 eV	
	10^−3^α	*d_p_*	10^−3^α	*d_p_*	10^−3^α	*d_p_*	10^−3^α	*d_p_*	10^−3^α	*d_p_*
**Si**	36.69	313	20.72	555	15.03	765	12.30	935	3.74	3072
**Ge**	613.71	19	606.03	19	597.95	19	558.55	21	152.73	75
**GaP**	10.11	1137	2.18	5275	0.94	12273	0.52	22115	0	-
**GaAs**	199.61	58	124.46	92	92.48	124	79.61	144	38.20	301
**GaSb**	605.83	19	588.26	20	487.41	24	436.25	26	213.52	54
**InP**	186.49	62	139.28	83	113.08	102	101.49	113	59.99	192
**InAs**	559.15	21	471.20	24	326.14	35	256.93	45	118.38	97
**InSb**	570.78	20	563.85	20	556.95	21	504.57	23	360.12	32

**Table 2:** Absorption coefficient (α, cm^−1^) [45] and penetration depth (*d_p_*, nm) (calculated using Equation (5) of single crystal SiC and amorphous SiC for different laser wavelengths and corresponding photon energy (hυ (eV), where h is Planck’s constant).

Wavelength (nm)	hυ (eV)	Single Crystal SiC		Amorphous SiC	
		α	*d_p_*	α	*d_p_*
207	5.994	1.5 × 10^6^	8	1.5 × 10^6^	8
225	5.515	3.0 × 10^5^	38	1.5 × 10^6^	8
248	5.003	6.0 × 10^4^	192	1.2 × 10^6^	10
276	4.496	2.0 × 10^4^	575	1.0 × 10^6^	12
310	4.003	4.0 × 10^3^	2.875 × 10^3^	8.0 × 10^5^	14
354	3.505	1.6 × 10^3^	7.188 × 10^3^	5.0 × 10^5^	23
413	3.004	30	3.833 × 10^5^	4.0 × 10^5^	29
496	2.502	15	7.667 × 10^5^	3.0 × 10^5^	38
620	2.001	8.0	1.438 × 10^6^	1.6 × 10^5^	72
827	1.500	5.0	2.300 × 10^6^	5.0 × 10^4^	230
1240	1.001	5.0	2.300 × 10^6^	4.0 × 10^4^	288
